# Investigating the Effect of Rosemary Essential Oil, Supercritical CO_2_ Processing and Their Synergism on the Quality and Microbial Inactivation of Chicken Breast Meat

**DOI:** 10.3390/foods12091786

**Published:** 2023-04-25

**Authors:** Fabio Santi, Riccardo Zulli, Elisa Lincetti, Alessandro Zambon, Sara Spilimbergo

**Affiliations:** 1 Industrial Engineering Department, University of Padova, Via Marzolo 9, 35131 Padova, Italy; 2 Department of Civil, Chemical, Environmental, and Materials Engineering (DICAM), University of Bologna, Via Terracini 28, 40131 Bologna, Italy

**Keywords:** supercritical CO_2_, MAP, essential oils, *Escherichia coli*, *Listeria innocua*, mesophilic and psychrophilic microorganisms

## Abstract

Fresh chicken meat is a very perishable good, even at refrigerated storage conditions, due to psychrophilic microbial growth and physicochemical changes. The present study focuses on the use of rosemary (*Rosmarinus officinalis* L.) essential oil (REO), supercritical CO_2_ processing and their synergism to increase the microbial inactivation in chicken breast meat. *E. coli* and *L. innocua* were inoculated on the chicken breast surface, and the inactivation effects of two different processes, namely SC-CO_2_ and SC-MAPCO_2_, were compared with or without the addition of REO. Moreover, the impact of the treatments on the superficial color of the meat was considered. The study demonstrated a synergic effect with 1% REO and supercritical CO_2_ for the inactivation of *E. coli* on chicken meat, while for *L. innocua,* there was no synergism. Regarding SC-CO_2_ treatment, the *E. coli* reduction was 1.29 and 3.31 log CFU/g, while for *L. innocua*, it was 1.42 and 1.11 log CFU/g, respectively, without and with the addition of 1.0% of REO. The same amount of REO allowed us to obtain a reduction of 1.3 log CFU/g of *E. coli* when coupled with SC-MAPCO_2_. For *L. innocua*, no reduction was obtained, either with SC-MAPCO_2_ or together with REO. The synergism of SC-MAPCO_2_ with 1% REO was confirmed for the total psychrophilic bacteria, demonstrating a strong dependence on the microorganism. The color modification induced by the SC-MAPCO_2_ process was lower than the SC-CO_2_ treatment. Overall, this study demonstrated a possible synergism of the technologies which can support the development of innovative methods to improve the safety and shelf-life of chicken breast meat.

## 1. Introduction

Chicken breast meat is a product consumed worldwide. The world production of chicken meat is growing and growing, and it is expected to reach 139.19 million tons per year in 2025 [1]. Chicken breast meat is highly consumed thanks to its low cost, versatility and quick preparation, and for the absence of religious restrictions related to its consumption [2,3]. Chicken meat has better nutritional characteristics compared to other types of meat. In particular, if compared with red meat, it has a lower fat content (especially saturated fats), a higher content of proteins, and a lower caloric content [4]. For these reasons, it is recommended for people who want to reduce their fat intake or those who suffer from coronary heart diseases [4].

Fresh chicken meat is very perishable, despite refrigerated storage, due to psychotropic microbial growth and physicochemical changes [5]. The presence and availability of several nutrients such as proteins, free amino acids, vitamins, fats, mineral salts, and moisture allow for the survival and growth of different microorganisms throughout the whole production and distribution chain. The main step that enhances the possibility of microbial contamination and colonization of the muscle tissues is the slaughtering process, during which the microorganisms normally present in the gastrointestinal tract, skin, lungs and feathers might contaminate the meat [3]. Outbreaks associated with the incorrect handling and the presence of pathogenic microorganisms in chicken meat, such as *Escherichia coli* O157:H7, *Salmonella* spp., *Campylobacter* spp. and *Listeria monocytogenes*, are very common [3,6]. A recent multi-country outbreak of *Salmonella* Mbandaka ST413 linked with the consumption of chicken was reported by the EFSA (European Food Safety Authority). From September 2021 to November 2022, this epidemic led to 196 cases in different European countries (Czechia, Estonia, Finland, France, Germany, Ireland, Netherlands, United Kingdom and Israel), among which were 19 hospitalizations, five cases of septicemia and one death [7]. Current technologies to increase the safety of raw chicken meat are still limited. Indeed, the use of heat, which is the most effective technique to kill microorganisms, cannot be used for the raw/chilled food segment. For this reason, research on low-temperature technologies has been focused on developing innovative processes to improve the safety and shelf-life of raw chicken meat without using high temperatures. Some innovative techniques such as ultrasounds (US) [8], UV-C irradiations [6] and gamma-irradiations [9] have been studied on chicken meat. However, these techniques have some disadvantages. US usually leads to the formation of free radicals, and its industrialization has some limitations due to the high investment costs and lack of regulative agreements [8]. UV-C, instead, might have negative effects such as off-flavor, browning, texture breakdown, damage and cell malfunction on meat [10]. Gamma-irradiations, instead, are effective for meat treatment and are already authorized by the FDA (Food and Drug Administration); however, this technique still has strict regulations in Europe and cannot yet be used for the majority of food products. Another obstacle to the industrialization of this method is that consumers frequently show aversion towards food irradiation [10]. Moreover, the implementation of this technique needs both high investment costs and measures for the operators’ safety [11]. Another technique that might be used to improve the shelf-life of chicken meat is high hydrostatic processing (HHP), which uses high pressures (>300 MPa) to inactivate microorganisms and has been demonstrated to be effective on chicken breast meat, especially against *L. monocytogenes*, *E. coli* and *S. typhimurium* [12]. However, this technology has strong impacts on structural, physiochemical, morphological and textural characteristics of the meat. In particular, an increase in the product hardness, cohesiveness, gumminess and chewiness has been observed, together with a strong lipid oxidation induced by pressures higher than 450 MPa [12].

Supercritical carbon dioxide (SC-CO_2_) treatment has been studied as an alternative low-temperature method to inactivate microorganisms on different food matrices [3,13,14,15] at relatively low pressure, compared to HHP. Recently, a new method for supercritical food inactivation using modified atmosphere packaging (MAP) with a high concentration of CO_2_ (SC-MAPCO_2_) has been developed by Spilimbergo et al. [16]. SC-MAPCO_2_ consists of pressurizing products already packaged in a CO_2_-rich atmosphere, avoiding any product handling after the process, thus reducing the risk of post-process contamination. In this way, the gas inside the packaging reaches the supercritical condition, exerting its antimicrobial power during the treatment.

This new method has been demonstrated to be effective at lab scale on fresh-cut carrots and coconut and coriander, obtaining inactivation results very close to the SC-CO_2_ method, but with milder effects on the product aspect [17,18]. This new method has never been studied on animal-origin products, but SC-CO_2_ has been demonstrated to be effective in the inactivation of microorganisms on meat products. Therefore, there is interest in understanding the potential of this new decontamination technology and its comparison with SC-CO_2_ in terms of quality retention and microbial inactivation. Bae et al. [19], for instance, obtained 1.69 log CFU/cm^2^ of inactivation for mesophilic bacteria by treating fresh pork meat at 12 MPa and 40 °C for 30 min. Chicken breast meat has been treated with SC-CO_2_, thereby obtaining 3.96 log CFU/g of inactivation of inoculated *E. coli* after a 45 min treatment at 14 MPa and 45 °C [3]. The method has also been coupled with antimicrobial substances, especially essential oils, such as rosemary and coriander, in a proof-of-concept study obtaining promising results in terms of inactivation [3]. However, little is known about the synergistic effect of SC-CO_2_ and antimicrobial agents including essential oils, but it can hypothesized that an increment of the inactivation can be achieved by choosing a correct amount of oil.

In this context, several studies have shown the potential to improve the preservation of different food products by coupling innovative inactivation techniques [20] with antimicrobial substances such as essential oils [3], spice extracts [21] and bacteriocins [22]. In a recent paper, Chen et al. [23] applied HHP on chicken meat pretreated with papaya extract to inactivate inoculated *Salmonella*, obtaining an inactivation of 6 log CFU/g. Stratakos et al. [24], instead, explored the possible synergism between HHP and a packaging activated with 10% of a coriander essential oil solution in ethanol to inactivate *Listeria monocytogenes* in ready-to-eat chicken meat.

Among these substances, the antimicrobial and antifungal activity of rosemary essential oil (REO, *Rosmarinus officinalis* L.) has been extensively demonstrated against different microorganisms, such as different strains of *E. coli* [25], and in different matrices such as soft cheese [26] and broccoli florets [27]. Moreover, its synergic effect with SC-CO_2_ has been studied and demonstrated effective on raw almonds [13].

The present study aims to investigate the effect of REO, CO_2_-based processing (SC-CO_2_ and SC-MAPCO_2_) and their synergism in the treatment of raw chicken meat. Results were focused on the microbial inactivation capacity against inoculated *E. coli* and *L. innocua* at different REO concentrations, following a 3 × 4 design of experiment. Additionally, the effect of the treatments on the qualitative aspects in terms of color, pH and water activity was evaluated.

## 2. Materials and Methods

### 2.1. Culture and Cell Suspension

*Escherichia coli* NCTC 9001 and *Listeria innocua* NCTC 11288 strains were used for the inoculation of the samples. The cultures’ preparation methods are described, respectively in [3] and in [28], with some modifications. Briefly, the *E. coli* and *L. innocua* cultures were incubated overnight in Luria–Bertani (LB) medium broth (Lennox, Sacco System, Como, Italy) and in BHI Broth (Microbiol diagnostici, Cagliari, Italy) at 37 °C, respectively. The microbial suspensions were centrifuged (Rotina 380 R, Hettich, Tuttlingen, Germany) at 6000 rpm for 8 min, the supernatant was removed, and the pellet was resuspended in Ringer’s solution (Merck, Darmstadt, Germany) in order to reach a final concentration of 10^8^ CFU/mL.

### 2.2. Sample Preparation and Microbial Inoculation

The chicken breasts used in this study were purchased from a local market in Padova (Italy) and processed on the same day to ensure freshness. Similar squared chicken breast pieces weighing 5 ± 0.05 g were prepared under a laminar flow cabinet to minimize contamination. Samples were used without further manipulation for the detection of natural microflora and for physicochemical analyses on untreated samples. For the inactivation experiments with *E. coli* and *L. innocua*, 100 µL of the microbial suspension was added to the chicken pieces cubes and left for 15 min at room temperature under the laminar flow cabinet. For the treatment with rosemary (*Rosmarinus officinalis* L.) essential oil (REO) (Erbamea, Perusa, Italy), the chicken cubes were sprinkled with different percentages (0.1, 0.5, and 1.0% volume/weight) of REO and left for 15 min at room temperature under the laminar flow cabinet. The samples were either analyzed directly as controls or processed before analysis.

### 2.3. High-Pressure Processes

Two high-pressure processes were investigated and compared. The first one (SC-CO_2_), previously described by González-Alonso et al. [3], treats the samples by direct contact with supercritical CO_2._ Briefly, a sample was inserted in a stainless steel vessel, which was then closed, filled and pressurized with CO_2_ (Nippon gasses, carbon dioxide 4.0, Milan, Italy). After the desired pressure was reached, it was maintained for a specific holding time. The second process (SC-MAPCO_2_), previously described by Barberi et al. [17], is instead performed on samples inserted in a high-gas barrier, multilayer (PA/EVOH/PA/PE) film (Euralpack, Schoten, Belgium) filled with CO_2_ and pressurized in a water-driven plant. The packaging material was selected thanks to its low CO_2_ permeability (<6.5 cm^3^/m^2^/d/bar) and its resistance to high pressure. The volume of the plastic bags was fixed at 100 ± 10 mL. To study the possible synergic effects between the two methods with and without REO, process conditions were chosen to guarantee a significant but not complete inactivation of *E. coli* and *L. innocua* when REO was not used; in particular, 14 MPa and 40 °C for 15 min were selected. Moreover, in applications with REO, temperature should not exceed 40 °C to avoid thermal degradation of the oil [29]. In both treatments, the desired pressure was reached in approximately 2 min, while the depressurization was almost instantaneous.

### 2.4. Microbial Enumeration

Each sample was placed in a sterile 50 mL falcon tube, to which 45 mL of sterile Ringer’s solution was added. The tube was vortexed (ZX3 Advanced Vortex Mixer, Velp Scientifica, Usmate Velate, Italy) at 2200 rpm for 90 sec. Successively, this solution was serially diluted (1:10) in Ringer’s solution. For the enumeration of *E. coli* [30] and *L. innocua* [28], 100 μL of the selected dilutions were spread-plated on MacConkey agar with crystal violet (Microbiol diagnostici, Cagliari, Italy) and BHI agar (Microbiol diagnostici, Cagliari, Italy), respectively. Plates were incubated for 24 h at 37 °C in an incubator (Memmert, Schwaback, Germany) and then enumerated. For mesophilic and psychrophilic natural microflora, non-inoculated samples were similarly analyzed by pour-plating in plate count agar (PCA, Microbiol diagnostici, Cagliari, Italy), in 1 mL of solution. The plates were then incubated at 30 °C for 72 h and at 10 °C for 120 h, for mesophilic and psychrophilic bacteria, respectively. Results are expressed as log CFU/g.

### 2.5. Physicochemical Analysis: Color, pH and a_w_

The surface color of the samples was measured using a Tristimulus colorimeter (NR100, 3nh, Guangzhou, China) in the CIE 1976 (L*, a*, b*) color space. The color modification caused by treatments was expressed as total color change (∆E) according to Equation (1) [31]:

(1) $$\Delta E\;=\sqrt{{\left(\Delta a^{*}\right)}^{2}+{\left(\Delta b^{*}\right)}^{2}+{\left(\Delta L^{*}\right)}^{2}}$$

where L* is the lightness index (100 for white to 0 for black), a* is the redness index (red when positive to green when negative), and b* is the yellowness index (yellow when positive to blue when negative).

The pH was determined with a pH meter (pH1100, VWR, Leuven, Belgium) with an electrode for solid samples (spear 220, VWR, Leuven, Belgium), while a_w_ was measured with an a_w_ meter (HygroPalm HP23-AW-A, Bassersdorf, Switzerland).

Each measurement was performed at least in triplicate.

### 2.6. Design of Experiment and Statistical Analysis

The possible synergism between the treatments and the REO concentration was evaluated by a randomized 3 × 4 design of experiment to analyze the effect on three different response variables: color modification and *E. coli* and *L. innocua* inactivation. Specifically, the processing method consists of three levels: control, SC-CO_2_ and SC-MAPCO_2_, while REO concentration has four levels: 0, 0.1, 0.5 and 1.0%.

Statistical analyses were performed in Minitab^®^. Mean values were used to compare differences between treatments. The existence of significant differences (α = 0.05) between different treatments was studied with an ANOVA and pair comparison within a group with its post hoc analysis (Tukey HSD).

## 3. Results

### 3.1. Comparison between SC-CO_2_ and SC-MAPCO_2_ without REO

**Figure 1:**
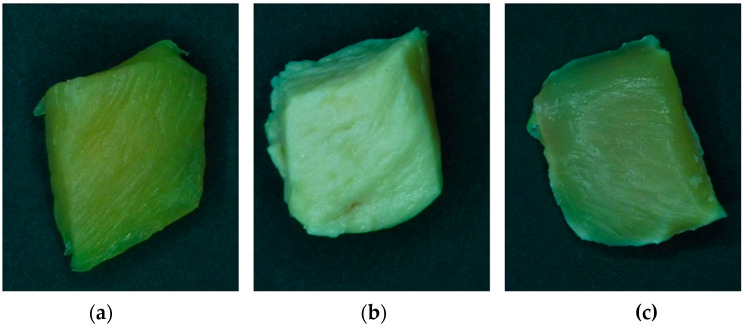
Pictures of raw (**a**), treated with SC-CO_2_ (**b**) and treated with SC-MAPCO_2_ (**c**) chicken breast samples. Treatment conditions: 14 MPa, 40 °C, 15 min.

**Figure 2:**
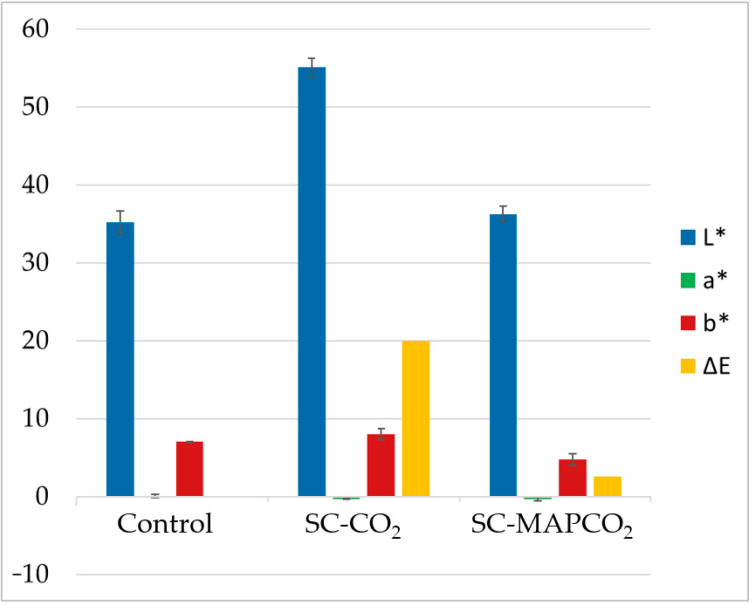
CIELAB color parameters (L*, a* and b*) and total color difference (ΔE) with respect to the raw chicken, for raw (Control), treated with SC-CO_2_, and treated with SC-MAPCO_2_ samples.

Chicken breast samples were treated with SC-CO_2_ and SC-MAPCO_2_ at the following process conditions: 14.0 MPa, 40 °C and 15 min. Pressure and temperature were chosen in accordance with Gonzáles-Alonso et al. [3], while 15 min was chosen as the processing time, because a longer time (≥30 min) led to complete inactivation of *E. coli* when the samples were treated with SC-CO_2_ alone. Figure 1 shows the pictures of the raw untreated samples in comparison with the treated ones. The CIELAB color parameters (L*, a* and b*) and the total color difference between the treated and untreated samples (ΔE) are reported in Figure 2.

The SC-CO_2_ treatment led to significant modifications in the visual aspect of the chicken meat. The lightness parameter L* increased from 35.15 ± 2.98 to 55.07 ± 2.36, resulting in a total color difference equal to 19.94 and a cooked-like visual appearance. Similar results were also obtained by González-Alonso et al. [3], in whose study the chicken samples treated with SC-CO_2_ (40 °C, 45 min, 8 and 14 MPa) showed a significant increase in superficial lightness L*. Indeed, other studies on protein matrices, in particular shrimps [32], pork [19] and ground beef [33], showed important color modifications caused by the treatment.

These modifications may be caused, in accordance with [19,34], by the effect of high-pressure CO_2_ on the molecular interaction and conformation of proteins, which can lead to their denaturation. In particular, the process may cause the denaturation of myoglobin and the consequent release of heme groups and coagulation of myofibrillar proteins [35]. Moreover, Monhemi et al. [36] simulated the effect of SC-CO_2_ on the molecular response of two different proteins: myoglobin and lysozyme, concluding that the protein denaturation could be caused by the weakening of the hydrophobic interactions and therefore the integrity of the tertiary structure.

Regarding the SC-MAPCO_2_, the color change was less if compared with the SC-CO_2_ treatment, resulting in a ∆E lower than 3, meaning that the visual modification with respect to the non-treated product is not substantial [31].

In Table 1, pH and water activity (a_w_) values for non-treated (control), treated with SC-CO_2_ and treated with SC-MAPCO_2_ are reported. The treatments did not significantly change the pH of the chicken breast meat. The a_w_ of the treated samples is slightly lower than the control samples. This little change may be due to the loss of water by applying high pressures, which is confirmed by the weight reduction of the samples after the treatment, which was about 8% for SC-CO_2_ and 5.5% for SC-MAPCO_2_.

In order to compare the microbial inactivation efficiency of the two methods, two fecal contamination indicators, *Escherichia coli* and *Listeria innocua*, were used as test microorganisms for the challenge test. In this work, the strains *E. coli* NCTC 9001 and *L. innocua* NCTC 11288, were used as surrogates of the pathogenic strains *E. coli* O157:H7 and *L. monocytogenes* [37]. Table 2 reports the inactivation data.

Approximately 1.29 log CFU/g of *E. coli* and 1.42 log CFU/g of *L. innocua* were reduced by SC-CO_2_. The inactivation achieved for *E.coli* confirmed the data obtained by González-Alonso et al. [3] at the same process conditions on chicken breast meat. In another study, Morbiato et al. [38] obtained a similar inactivation, 1.76 ± 0.16 log CFU/g, for the *Salmonella enterica* on chicken breast meat at 40 °C and 10 MPa. In their case, the product was processed for supercritical drying, and the vessel was pressurized from 6 MPa up to 10 MPa at a rate of 0.4 MPa/min and then depressurized at a rate of 1 MPa/min.

Similar results have been achieved on other meat products. Bae et al. [19] obtained an inactivation of 2.00 log CFU/cm^2^ of *Salmonella typhimurium* and 1.99 log CFU/cm^2^ of *E. coli* O157:H7 by treating fresh pork meat at 12 MPa and 40 °C for 30 min.

The inactivation achieved for *L. innocua* is slightly higher than that achieved by Wei et al. [39]. Specifically, they reduced 85% of *L. monocytogenes* after 2 h at 13.7 MPa and 35 °C. The higher inactivation achieved for *L. innocua* might be caused by the higher temperature used, and also by the different resistance to the process due to the different strain used compared with Wei et al.

In the case of SC-MAPCO_2_, the treatment was able to slightly reduce the initial load; however, the difference in microbial content was not significant if compared with the untreated control sample. This result suggests a strong dependence on the treatment with the food matrix. Indeed, in the case of other food matrices such as carrot and coriander [17,18], the inactivation of inoculated *E. coli* on the sample surface was also found to be higher after the treatment with the SC-CO_2_ method compared to the SC-MAPCO_2_ method. However, the inactivation with SC-MAPCO_2_ was significant, suggesting a better inactivation capacity of the treatment for vegetables than meat. In the case of meat, the presence of fats and proteins could play a significant role in protecting microorganisms from high-pressure CO_2_ bactericidal action [40]. However, a deeper investigation of the inactivation mechanisms should be addressed to confirm any hypothesis, also including the effect on more food matrices.

The difference in microbial reduction within the two CO_2_ treatments could be attributed to the lower amount of CO_2_ used in the SC-MAPCO_2_ compared to the one in the SC-CO_2_. Indeed, considering the selected conditions (14 MPa and 40 °C), the CO_2_ density is about 628.65 kg/m^3^ [41]; thus, the CO_2_ contained in the reactor used in the SC-CO_2_ method (15 mL) is about 6.92 g, and in the case of SC-MAPCO_2_ is about 0.17 g (considering a volume of the pouch of 100 mL at ambient pressure). This difference in CO_2_ quantity between the two methods could also support the color modification results.

### 3.2. Effect of REO Alone and with CO_2_ Treatments

After comparing the two treatments alone, the effect of REO and the evaluation of a possible synergic effect together with the supercritical CO_2_ treatments was investigated. The effect of the oil was studied in terms of both color change and the inactivation capacity against *E. coli* and *L. innocua* for both methods, following a randomized 3 × 4 design of experiment.

Three REO concentrations were selected: 0.1, 0.5 and 1.0% according to previous works by González-Alonso et al. and Hamedo et al. [3,26].

The CIELAB color parameters and the total color difference for the untreated and treated samples with different REO concentrations are reported in Table 3.

The application of REO in the unprocessed samples (control) did not significantly change the superficial color of chicken breast; in fact, the values L*, a* and b* were not significantly different, and the ∆E values were lower than 1.5 for the entire range of REO concentrations used. Additionally, the results demonstrated that different percentages of REO did not further increase the color change in the case of SC-CO_2_ treated samples. On the contrary, REO caused an increment of the parameter L* and the ∆E after the SC-MAPCO_2_ process, but these values were still significantly lower than the ones achieved after the application of SC-CO_2_ treatment. Moreover, the color difference was not influenced by the concentration of the oil. Overall, these data confirm that the SC-MAPCO_2_ had a lower effect on the change of product color, even when REO was added.

The results regarding the Inactivation of the inoculated samples with *E. coli* and *L. innocua* are reported in Table 4.

The data obtained showed that the addition of REO in raw chicken meat (untreated samples) was not able to reduce *E. coli* and *L*. *innocua,* even at the highest concentration applied. A possible explanation may be the matrix composition, since proteins and fats are able to bind the volatile compounds of essential oils [42], becoming less effective than in non-protein matrices. Indeed, the antimicrobial effect of REO is mainly caused by volatile components such as 1,8-cineole, α-pinene, camphor, camphene, borneol, myrcene, bornyl acetate, terpineol, linalool, limonene and caryophyllene [27,43]. Our result is in accordance with the work by Hamedo et al. [26] on soft cheese, to which REO was similarly added. Different studies reported instead a significant inactivation of various microorganisms on fruits and vegetables after the application of essential oils. Zhang et al. showed the application of thyme essential oil on organic cantaloupes, obtaining a reduction of 2.26, 3.06 and 1.49 log CFU/cm^2^ for *E. coli* O157:H7, *S. enterica* and *L. monocytogenes*, respectively [44].

**Figure 3:**
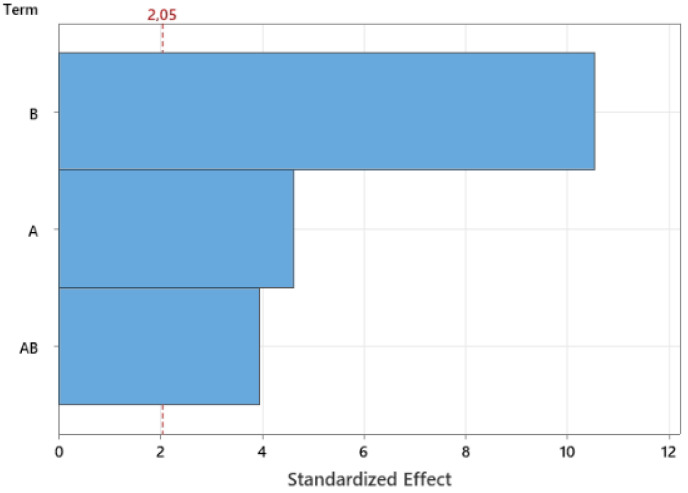
Pareto chart regarding the effect of the REO concentration (A), the processing method (B), and the combination of the two (AB) with respect to *E. coli* inactivation. The red dotted line indicated the minimum *t*-value at a confidence level of 95.0%.

In the case of *E. coli,* a synergic effect on the inactivation was observed when 1% REO was used in combination with high-pressure processing. In the case of SC-CO_2_, 1% REO caused an additional 2.12 log CFU/g reduction compared to the SC-CO_2_ alone. On the contrary, when a lower concentration of oil (0.1 and 0.5%) was used, no significant effect was achieved. A similar synergic effect was also observed for SC-MAPCO_2_. The inactivation became significantly different in comparison with the untreated control only for 1% REO, reaching a reduction of 1.3 log CFU/g. The effect of the oil and its synergism with the treatments was also confirmed by the Pareto chart reported in Figure 3, which shows that the process is the most influential parameter for inactivation, followed by the oil concentration, followed by the combination of the process and REO.

The synergism between SC-CO_2_ and REO for chicken breast meat was also investigated by González-Alonso et al. [3], who treated the inoculated samples at 14 MPa and 45 °C for 45 min. In that case, the only application of SC-CO_2_ led to an inactivation of 4.74 ± 1.05 log CFU/g of *E. coli* (ATCC 25922), and the addition of REO (1%) did not improve the process performance significantly. These results are in contrast with the ones obtained in this study. A possible explanation might be due to different strains of *E. coli* that are more sensitive to the process being used. Indeed, in this study, a complete inactivation was achieved after 30 min, while González-Alonso et al. were still able to count cells after 45 min of treatment.

The precise mechanism of the synergism between REO and high-pressure CO_2_ should be further investigated, including in the study the effect of different types of essential oils and different microorganisms. However, a possible explanation of the synergism might be already explained by the high solvating power and lipophilicity of supercritical CO_2_. Specifically, the good solubility of the oil volatile components in SC-CO_2_ can enhance their penetration through the bacterial membrane. A higher percentage of essential oil (more than 1%) might also have a stronger effect, but this may negatively influence the consumer acceptance of the process.

In this study, a strong dependence on the bacterial strain was also observed for the synergism. Specifically, in the case of *L. innocua,* REO was not able to increase the inactivation capacity of the two methods, even with the highest concentrations used. This difference with *E. coli* could be caused by the lower antimicrobial power of the oil against this strain, and not only by the membrane composition. Texeira et al. [45] observed a higher MIC (minimum inhibitory concentration) of REO on *L. innocua* compared with that of *E. coli*, and consequently a greater amount of REO may have been needed to obtain an antimicrobial effect. As a further demonstration of the dependence on the type of microorganism, some inactivation experiments for the natural present total mesophilic and total psychrophilic bacteria were performed using SC-MAPCO_2_ coupled with 1% of REO. The results are reported in Table 5.

Fresh chicken meat presented an initial microbial load equal to 5.38 and 5.24 log CFU/g of mesophilic and psychrophilic bacteria, respectively. Additionally, in this case, the only addition of 1% of REO did not significantly reduce the natural present bacteria load. SC-MAPCO_2_ alone was able to significantly reduce the total mesophilic bacteria of 1.28 log CFU/g, while the psychrophilic load was not different from the control. Adding 1% REO caused a reduction of both mesophilic and psychrophilic bacteria. The synergism was significant only for the psychrophilic bacteria.

This is an interesting result, since in the work of Gonzales-Alonso et al. [3], a mesophilic microbial reduction of 2.64 ± 0.32 log CFU/g was obtained with SC-CO_2_ coupled with fresh rosemary at the same pressure and temperature conditions, but after 45 min of treatment. These findings are promising for the obtainment of a prolonged shelf-life of the product; however, further data are needed to confirm this hypothesis.

## 4. Conclusions

This work studied the effect of REO and two CO_2_-based processes on the microbial inactivation and retention of qualitative aspects of raw chicken breast meat. The inactivation capacity was evaluated for inoculated *E. coli* and *L. innocua*. The SC-MAPCO_2_ method was able to better maintain the visual aspect of the product compared with SC-CO_2_. On the contrary, the inactivation capacity was significant only for the SC-CO_2_ treatment for both microorganisms. The REO alone was not able to induce any significant reduction of inoculated *E. coli* and *L. innocua*. When REO was used in a synergistic manner with SC-CO_2_ at 1%, the inactivation was higher compared with SC-CO_2_ alone, but only for *E. coli*. Similarly, a significant inactivation capacity compared to the control samples is possible with SC-MAPCO_2_, but only in synergism with 1% REO. In this case, the inactivation is significant only for *E. coli* and the total psychrophilic bacteria, but not significant for *L. innocua* and the total mesophiles.

Regarding the color change, SC-CO_2_ has a strong impact on L*, a* and b* while SC-MAPCO_2_ has a mild effect. However, when REO was added to the SC-MAPCO_2_ process, the color change became significantly different for L*, compared to the untreated samples with a higher value of the ΔE. In any case, the overall color change was still lower compared to SC-CO_2_.

Overall, the results demonstrated the possible synergism between the SC-CO_2_/SC-MAPCO_2_ at a specific percentage of REO, but also a strong dependence on the type of microorganism investigated. Future studies should focus on the possible synergisms with different types of EO and microbial strains. Moreover, the effect of the process and REO on the sensorial and quality attributes of the product should be studied for further application at industrial level, as well as for their effect on the possible extension of storage time.

## Figures and Tables

**Table 1:** pH and water activity of samples non-treated (control), treated with SC-CO_2_ and treated with SC-MAPCO_2_. Means with different superscript letters are significantly different (*p* < 0.05).

Sample	pH	a_w_
Control	5.85 ± 0.05 ^a^	0.971 ± 0.002 ^a^
SC-CO_2_	5.90 ± 0.09 ^a^	0.951 ± 0.003 ^b^
SC-MAPCO_2_	5.72 ± 0.07 ^a^	0.954 ± 0.007 ^b^

**Table 2:** *E. coli* and *L. innocua* load, expressed in log CFU/g, on the inoculated samples non-treated (control), treated with SC-CO_2_ and treated with SC-MAPCO_2_. Means with different superscript letters are significantly different (*p* < 0.05).

Sample	*E. coli* (log CFU/g)	*L. innocua* (log CFU/g)
Control	7.03 ± 0.06 ^a^	7.41 ± 0.14 ^a^
SC-CO_2_	5.74 ± 0.62 ^b^	5.99 ± 0.11 ^b^
SC-MAPCO_2_	6.40 ± 0.21 ^ab^	7.25 ± 0.06 ^a^

**Table 3:** CIELAB color parameters (L*, a* and b*) and total color difference (ΔE) with respect to the raw chicken, for raw (control), treated with SC-CO_2_, and treated with SC-MAPCO_2_, sprinkled with different percentages of REO. Means with different superscript letters in the same column are significantly different (*p* < 0.05).

	REO (%)	L*	a*	b*	∆E
Control	0.0	35.15 ± 2.98 ^c^	0.06 ± 0.33 ^a^	7.03 ± 0.06 ^a^	/
	0.1	33.93 ± 0.53 ^c^	−0.02 ± 0.48 ^a^	6.50 ± 0.26 ^abc^	1.33
	0.5	33.93 ± 1.06 ^c^	0.14 ± 0.26 ^a^	6.97 ± 0.14 ^ab^	1.22
	1.0	33.72 ± 1.49 ^c^	0.29 ± 0.50 ^a^	6.93 ± 0.01 ^ab^	1.45
SC-CO_2_	0.0	55.07 ± 2.36 ^a^	−0.31± 0.16 ^a^	7.96 ± 1.49 ^a^	19.95
	0.1	52. 29 ± 1.02 ^a^	−0.08 ± 0.78 ^a^	6.26 ± 2.53 ^ab^	17.16
	0.5	53.43 ± 2.15 ^a^	0.29 ± 0.51 ^a^	7.02 ± 0.20 ^ab^	18.28
	1.0	53.11 ± 0.81 ^a^	−0.30 ± 0.17 ^a^	5.79 ± 1.28 ^abc^	18.01
SC-MAPCO_2_	0.0	36.27 ± 2.06 ^c^	−0.33 ± 0.50 ^a^	4.74 ± 1.42 ^abc^	2.58
	0.1	42.84 ± 2.97 ^b^	−0.59 ± 0.70 ^a^	2.53 ± 0.77 ^c^	8.83
	0.5	42.56 ± 0.26 ^b^	−0.99 ± 0.35 ^a^	3.94 ± 1.05 ^bc^	8.10
	1.0	43.26± 2.13 ^b^	−0.99 ± 0.31 ^a^	3.66 ± 0.74 ^bc^	8.84

**Table 4:** *E. coli* and *L. innocua* concentration, expressed in log CFU/g, for raw (control), treated with SC-CO_2_, and treated with SC-MAPCO_2_, sprinkled with different percentages of REO. Means with different superscript letters are significantly different (*p* < 0.05).

	REO (%)	*E. coli* (log CFU/g)	*L. innocua* (log CFU/g)
Control	-	7.03 ± 0.06 ^a^	7.41 ± 0.14 ^a^
	0.1	6.50 ± 0.26 ^abc^	7.50 ± 0.11 ^a^
	0.5	6.97 ± 0.14 ^ab^	7.30 ± 0.06 ^a^
	1.0	6.93 ± 0.01 ^ab^	7.48 ± 0.25 ^a^
SC-CO_2_	-	5.74 ± 0.62 ^cde^	5.99 ± 0.11 ^c^
	0.1	5.02 ± 0.08 ^de^	6.44 ± 0.13 ^bc^
	0.5	4.58 ± 0.25 ^ef^	6.31 ± 0.01 ^c^
	1.0	3.62 ± 0.26 ^f^	6.30 ± 0.62 ^c^
SC-MAPCO_2_	-	6.40 ± 0,21 ^abc^	7.25 ± 0.06 ^a^
	0.1	6.56 ± 0.36 ^abc^	6.96 ± 0.06 ^ab^
	0.5	5.98 ± 0.10 ^bc^	7.02 ± 0.05 ^ab^
	1.0	5.73 ± 0.74 ^cd^	7.19 ± 0.22 ^a^

**Table 5:** Mesophilic and psychrophilic microbial population, expressed as log CFU/g, of fresh chicken breast (control) and that treated with SC-MAPCO_2,_ with and without REO (1%). Means with different superscript letters are significantly different (*p* < 0.05).

Sample	Mesophilic Bacteria	Psychrophilic Bacteria
Control	5.38 ± 0.04 ^a^	5.24 ± 0.30 ^a^
Control + 1% REO	5.12 ± 0.17 ^ab^	5.52 ± 0.47 ^a^
SC-MAPCO_2_	4.10 ± 0.66 ^bc^	4.56 ± 0.15 ^a^
SC-MAPCO_2_ + 1% REO	3.27 ± 0.62 ^c^	3.19 ± 0.70 ^b^

## Data Availability

The data are available from the corresponding author.
